# Long-term results of ulnar and radial reconstruction with interpositional grafting using the deep inferior epigastric artery for chronic hand ischemia

**DOI:** 10.1038/s41598-021-02530-6

**Published:** 2021-11-30

**Authors:** Hee Chang Ahn, Se Won Oh, Jung Soo Yoon, Seong Oh Park

**Affiliations:** 1 Department of Plastic and Reconstructive Surgery, CHA University Bundang Medical Center, Seongnam-si, Gyeonggi-do Korea; 2 Department of Plastic and Reconstructive Surgery, Hanyang University College of Medicine, Seoul, Korea; 3 Department of Plastic and Reconstructive Surgery, Dongguk University Medical Center, Goyang-si, Gyeonggi-do Korea

**Keywords:** Rheumatology, Risk factors, Rheumatic diseases, Autoimmune diseases

## Abstract

Chronic hand ischemia causes cold intolerance, intractable pain, and digital ulceration. If ischemic symptoms persist despite pharmacologic treatments, surgical interventions should be considered. This retrospective study evaluated the long-term results after ulnar and radial reconstruction using an interpositional deep inferior epigastric artery (DIEA) graft combined with periarterial sympathectomy. Patients who underwent this surgery from March 2003 to February 2019 were included. To evaluate variables influencing recurrence after the procedure, patients were divided into the recurred and non-recurred groups and their data were compared. Overall, 62 cases involving 47 patients were analyzed (16 and 46 cases in the recurred and non-recurred groups, respectively). The median DIEA graft length was 8.5 cm. The rates of rheumatic disease and female patients were significantly higher in the recurred than in the non-recurred group, without significant between-group differences in postoperative complication rates. In the multivariate analysis, underlying rheumatic disease and graft length had significant effects on recurrence. In Kaplan–Meier analysis, the 5- and 10-year symptom-free rates were 81.3% and 68.0%, respectively, with lower rates for cases with rheumatic disease. Thus, arterial reconstruction using an interpositional DIEA graft provides long-term sustainable vascular supply in patients with chronic hand ischemia, especially in those without rheumatic disease.

## Introduction

Chronic hand ischemia, represented by Raynaud’s phenomenon, causes cold intolerance, intractable pain, and digital ulceration^1,2^. Since these symptoms have detrimental effects on a patient’s daily life, occupation, and quality of life, appropriate treatment is essential. Initial treatments consist of avoidance of cold exposure, smoking cessation, and wearing warm gloves for protection. If symptoms are sustained or worsen, pharmacologic treatment with topical nitroglycerine, calcium channel blockers, tricyclic antidepressants, selective serotonin reuptake inhibitors, vasodilator drugs, or rheologic agents can be performed^3,4^. Recently, botulinum toxin A and fat grafting have also been reported as novel therapeutic modalities^5–7^. However, if symptoms persist or worsen despite these treatment approaches, a surgical intervention should be considered.

There are two main types of surgical treatments for chronic hand ischemia. The first is a periarterial sympathectomy, which removes sympathetic nerve input to the artery^8–12^. The second is an arterial reconstruction, which repairs or bypasses the occluded arterial segment^13,14^. These two methods can be complementary; however, in severely occluded cases, a periarterial sympathectomy alone is insufficient. In a recent systematic review, arterial reconstruction using an arterial bypass showed better results, including improved pain relief and wound healing, compared to periarterial sympathectomy alone^15^. The authors also determined that arterial reconstruction is necessary in selected cases and, therefore, we applied this technique^4^. In contrast, venous arterialization is another option; however, it is not a commonly used surgical technique^16,17^.

The most commonly used method for arterial reconstruction is bypass grafting using a vein^18–21^. The cephalic, brachial, and saphenous veins are commonly used as graft sources. In addition, polytetrafluoroethylene (PTFE) can be used when the autologous vein is inappropriate^15^. In contrast, the authors have performed arterial reconstructions using interpositional arterial grafts with the deep inferior epigastric artery (DIEA) instead of venous grafts. This study aimed to evaluate the long-term results of our surgical technique, which involves an interpositional DIEA graft combined with periarterial sympathectomy, in patients with symptoms of chronic hand ischemia, including intractable pain, fingertip ulceration, and necrosis.

## Results

### Results in the total study population

A total of 62 cases in 47 patients (15 patients; bilateral hands) were included. The mean age at the time of operation was 48.2 ± 13.3 years, and the median follow-up duration was 51.0 months. Among the total cases, 14 (22.6%) were of workers who were exposed to vibrating hand-held tools, and 42 (67.7%) were of patients with rheumatic disease. Systemic sclerosis (SSc) was the most common underlying condition (16 cases, 25.8%), with SSc and systemic lupus erythematous (SLE) being the second most common (nine cases, 14.5%), followed by SLE (six cases, 9.7%).

The median duration from symptom onset to the operation was 60 months. All patients had intractable pain, with ulceration in 25 cases (40.3%). Twenty-eight patients (56 hands) had intractable pain in bilateral hands. Fifty-one cases (82.2%) had not received any other invasive treatment prior to arterial reconstruction, while periarterial sympathectomy had been performed in six cases (9.7%) (Table 1).

**Table 1:** Demographic Characteristics of All Study Cases.

Variables	Total (%)	Recurred group (%)	Non-recurred group (%)	*p*-value
No. of cases	62	16	46	
Bilateral hands	30 (48.4)	8 (12.9)	22 (35.5)	0.881
Unilateral hand	32 (51.6)	8 (12.9)	24 (38.7)	
Age, yr ± SD	48.2 ± 13.3	49.0 ± 16.3	47.9 ± 12.3	0.777
**Sex**				0.007
Male	15 (24.2)	0 (0.0)	15 (32.6)	
Female	47 (75.8)	16 (100.0)	31 (67.4)	
Follow-up period, mo †	51.0 (28.0–90.0)	34.0 (20.5–82.5)	54.5 (28.0–99.0)	0.154
**Occupation**				0.090
Hand worker ††	14 (22.6)	1 (6.3)	13 (28.3)	
Other	48 (77.4)	15 (93.8)	33 (71.7)	
Smoking	9 (14.5)	0 (0.0)	9 (19.6)	0.096
**Comorbidity**				
DM	5 (8.1)	1 (6.3)	4 (8.7)	1.000
HTN	7 (11.3)	1 (6.3)	6 (13.0)	0.666
Thyroid disease	6 (9.7)	3 (18.8)	3 (6.5)	0.172
Cardiovascular disease	3 (4.8)	0 (0.0)	3 (6.5)	0.562
Pulmonary disease	2 (3.2)	0 (0.0)	2 (4.4)	1.000
Rheumatic disease	42 (67.7)	14 (87.5)	28 (60.9)	0.050
**Subtype of rheumatic disease**				0.192
SSc	16 (25.8)	8 (50.0)	8 (17.4)	
SSc + SLE	9 (14.5)	2 (12.5)	7 (15.2)	
SLE	6 (9.7)	3 (18.8)	3 (6.5)	
Mixed connective tissue disease	4 (6.5)	1 (6.3)	3 (6.5)	
RA	2 (3.2)	0 (0.0)	2 (4.4)	
Polymyositis	1 (1.6)	0 (0.0)	1 (2.2)	
Buerger's disease	2 (3.2)	0 (0.0)	2 (4.4)	
Sjögren syndrome	1 (1.6)	0 (0.0)	1 (2.2)	
RA + Buerger's disease	1 (1.6)	0 (0.0)	1 (2.2)	
Symptom onset-to-operation period, mo †	60.0 (20.0–120.0)	108.0 (54.0–186.5)	36.5 (8.0–100.0)	0.011
Intractable pain	62 (100.0)	16 (100.0)	46 (100.0)	1.000
Ulceration	25 (40.3)	6 (37.5)	19 (41.3)	0.789
Bilateral involve symptom (pain)	56 (90.3)	15 (93.8)	41 (89.1)	1.000
**Previous invasive treatment**				0.021
Periarterial sympathectomy	6 (9.7)	5 (31.3)	1 (2.2)	
Carpal tunnel release	2 (3.2)	0 (0.0)	2 (4.4)	
Vein graft	1 (1.6)	0 (0.0)	1 (2.2)	
PTA	1 (1.6)	0 (0.0)	1 (2.2)	
Sympathectomy + Vein graft	1 (1.6)	0 (0.0)	1 (2.2)	

SD, standard deviation; DM, diabetes mellitus; HTN, hypertension; SLE, systemic lupus erythematosus; RA, rheumatoid arthritis; PTA, percutaneous transluminal angioplasty.

^†^ Median, mo (interquartile range).

^††^ Hand workers refer to patients with jobs that have high exposure to vibrating hand-held tools, such as miners, construction workers, or mechanics.

### Comparisons between the recurred and non-recurred groups

Among the 62 total cases, there were 16 (25.8%) and 46 cases (74.2%) in the recurred and non-recurred group during the follow-up period. There were significantly more women (16 cases, 100.0%) in the recurred than in the non-recurred group (*p* = 0.007). In addition, the recurrence rates were significantly higher in patients with (14 cases, 87.5%) than in those without rheumatic disease (*p* = 0.05). There were no significant differences in the subtypes of rheumatic disease between the two groups.

The median time period between symptom onset and surgery was 108.0 months (interquartile range, 54.0–186.5) in the recurred group, which was significantly longer than that in the non-recurred group (*p* = 0.05). Moreover, preoperative invasive treatments were significantly different between the two groups (*p* = 0.021), with periarterial sympathectomy being more common in the recurred group (Table 1).

Recipient vessels for grafts consisted of the following four subtypes: the ulnar artery in Guyon’s canal, the distal ulnar artery to the superficial palmar arch or the common digital artery, the distal radial artery to the deep palmar arch of the princeps pollicis artery, or both (radial and ulnar arteries). Of the total cases, 55 (88.7%) involved the ulnar artery, which was the most common, with six (9.7%) involving the radial artery and one involving both the ulnar and radial arteries. The median DIEA graft length was 8.5 cm in all study cases, and an advanced periarterial sympathectomy involving the common and proper palmar digital arteries was performed in four cases (6.5%) (Table 2).

**Table 2:** Operation-related Variables in the Recurred and Non-recurred Groups.

Variables	Total (%)	Recurred group (%)	Non-recurred group (%)	*p*-value
**Operation side**				0.860
Left	38 (61.3)	10 (62.5)	28 (60.9)	
Right	24 (38.7)	6 (37.5)	18 (39.1)	
Bilateral operation	30 (48.4)	8 (26.7)	22 (73.3)	1.000
**Graft Type (recipient vessel)**				0.339
Ulnar artery in Guyon’s canal	6 (9.7)	1 (6.3)	5 (10.9)	
Ulnar artery to supf. palmar arch or common digital artery	49 (79.0)	12 (75.0)	37 (80.4)	
Radial artery to deep palmar arch of princeps pollicis artery	6 (9.7)	2 (12.5)	4 (8.7)	
Both (radial & ulnar artery)	1 (1.6)	1 (6.3)	0 (0.0)	
Length of graft, cm †	8.5 (7.0–10.0)	9.0 (8.0–10.0)	8.0 (7.0–10.0)	0.530
**Sympathectomy level**				0.272
Ulnar or radial artery to palmar arch	58 (93.6)	14 (87.5)	44 (95.7)	
Including CPDA & PPDA	4 (6.5)	2 (12.5)	2 (4.4)	
Concomitant amputation or debridement	2 (3.2)	2 (12.5)	0 (0.0)	0.064

CPDA, common palmar digital artery; PPDA, proper palmar digital artery.

^†^ Median (interquartile range).

While all cases in both groups showed postoperative improvements in pain, improvements in ulceration were significantly higher in the non-recurred than in the recurred group (*p* = 0.031) (Supplementary Fig. S1). The mean duration from surgery to symptomatic recurrence was 46.6 months. Eleven cases (17.7%) had recipient site complications, and nine cases (14.5%) had donor site complications in the overall study population (Table 3). There were no significant differences in the rates of postoperative complications between the two groups.

**Table 3:** Result-related Variables and Postoperative Complications in the Recurred and Non-recurred Groups.

Variables	Total (%)	Recurred group (%)	Non-recurred group (%)	*p*-value
Pain relief	62 (100.0)	16 (100.0)	46 (100.0)	
**Resolution of ulcer (n = 25)**				0.031
Improved	21 (84.0)	3 (50.0)	18 (94.7)	
No change	3 (12.0)	2 (33.3)	1 (5.3)	
Worsening	1 (4.0)	1 (16.7)	0 (0.0)	
Time to ulcer healing, wks (n = 21) †	4.0 (4.0–8.0)	4.0 (0.0–8.0)	4.5 (4.0–8.0)	0.426
Time to symptomatic recur, mo ± SD (n = 16)		46.6 ± 46.7		
**Recipient site complication**				0.721
SSI	4 (6.5)	0 (0.0)	4 (8.7)	
Delayed wound healing (> 3 wks)	7 (11.3)	2 (12.5)	5 (10.9)	
**DIEA donor site complications**				1.000
Infection	4 (6.5)	1 (6.3)	3 (6.5)	
Delayed wound healing (> 3 wks)	4 (6.5)	1 (6.3)	3 (6.5)	
Seroma	1 (1.6)	0 (0.0)	1 (2.2)	

SSI, surgical site infection; DIEA, deep inferior epigastric artery.

^†^ Median (interquartile range).

### Risk factor analysis for recurrence

In the univariate analysis, underlying thyroid disease (hazard ratio [HR], 3.983, 95% confidence interval [CI], 1.0169–14.835; *p* = 0.039), a previous sympathectomy (HR, 6.091, 95% CI, 2.063–17.983; *p* = 0.001), grafts to both the radial and ulnar arteries (HR, 22.553, 95% CI, 1.204–442.568; *p* = 0.037), and concomitant amputation (HR, 5.987, 95% CI, 1.299–27.588; *p* = 0.022) significantly affected recurrence. In the multivariate analysis, three factors showing significant effects on recurrence, including underlying rheumatic disease (HR, 11.242, 95% CI, 1.997–63.279; *p* = 0.006), sympathectomy involving the common palmar digital artery (CPDA) and the proper palmar digital artery (PPDA) (HR, 7.979, 95% CI, 1.473–43.209; *p* = 0.016), and graft length (HR, 1.472, 95% CI, 1.101–1.969; *p* = 0.009), were found to significantly affect patient outcomes (Table 4).

**Table 4:** Univariate and Multivariate Cox Regression Analyses of Risk Factors for Recurrence of Ischemic Symptoms.

Variables	Univariate analysis			Multivariate analysis		
	Unadjusted HR	95% CI	*p*-value	Adjusted HR	95% CI	p-value
Age, yr	1.012	0.973–1.053	0.558			
Sex (Male)	0	0	0.992			
Smoking	0	0	0.994			
**Comorbidity**						
DM	1.086	0.140–8.432	0.937			
HTN	0.486	0.064–3.685	0.485			
Thyroid disease	3.983	1.069–14.835	0.039			
Cardiovascular disease	0	0	0.994			
Pulmonary disease	0	0	0.994			
Rheumatic disease	4.032	0.912–17.821	0.066	11.242	1.997–63.279	0.006
Bilateral operation	0.917	0.342–2.463	0.864			
Bilateral symptoms	1.425	0.186–10.909	0.733			
**Occupation**						
Hard worker	0.136	0.018–1.042	0.055			
Other	Ref					
Symptom onset-to-operation period, mo	1.004	0.999–1.009	0.082			
Ulceration	1.341	0.472–3.813	0.582			
**Previous invasive treatment**						
Sympathectomy	6.091	2.063–17.983	0.001			
Carpal tunnel release	0	0	0.996			
Vein graft	0	0	0.997			
PTA	0	0	0.996			
Sympathectomy + Vein graft	0	0	0.998			
None	Ref					
**Graft Type (recipient vessel)**						
Ulnar artery in Guyon’s canal	Ref					
Ulnar artery to supf. Palmar arch or common digital artery	1.047	0.134–8.206	0.965			
Radial artery to deep palmar arch of princeps pollicis artery	1.943	0.176–1.470	0.588			
Both (radial & ulnar arteries)	22.553	1.204–422.568	0.037			
**Sympathectomy level**						
Ulnar or radial arteries to palmar arch	Ref			Ref		
Including CPDA & PPDA	3.271	0.724–14.772	0.123	7.979	1.473–43.209	0.016
Length of graft, cm	1.101	0.893–1.357	0.368	1.472	1.101–1.969	0.009
Concomitant amputation or debridement	5.986	1.299–27.588	0.022			
**Postop complication**						
SSI	0	0	0.994			
Delayed wound healing	1.945	0.411–9.209	0.402			
None	Ref					

PTA, percutaneous transluminal angioplasty; CPDA, common palmar digital artery; PPDA, proper palmar digital artery; SSI, surgical site infection; Ref, reference.

### Long-term results

**Figure 1:**
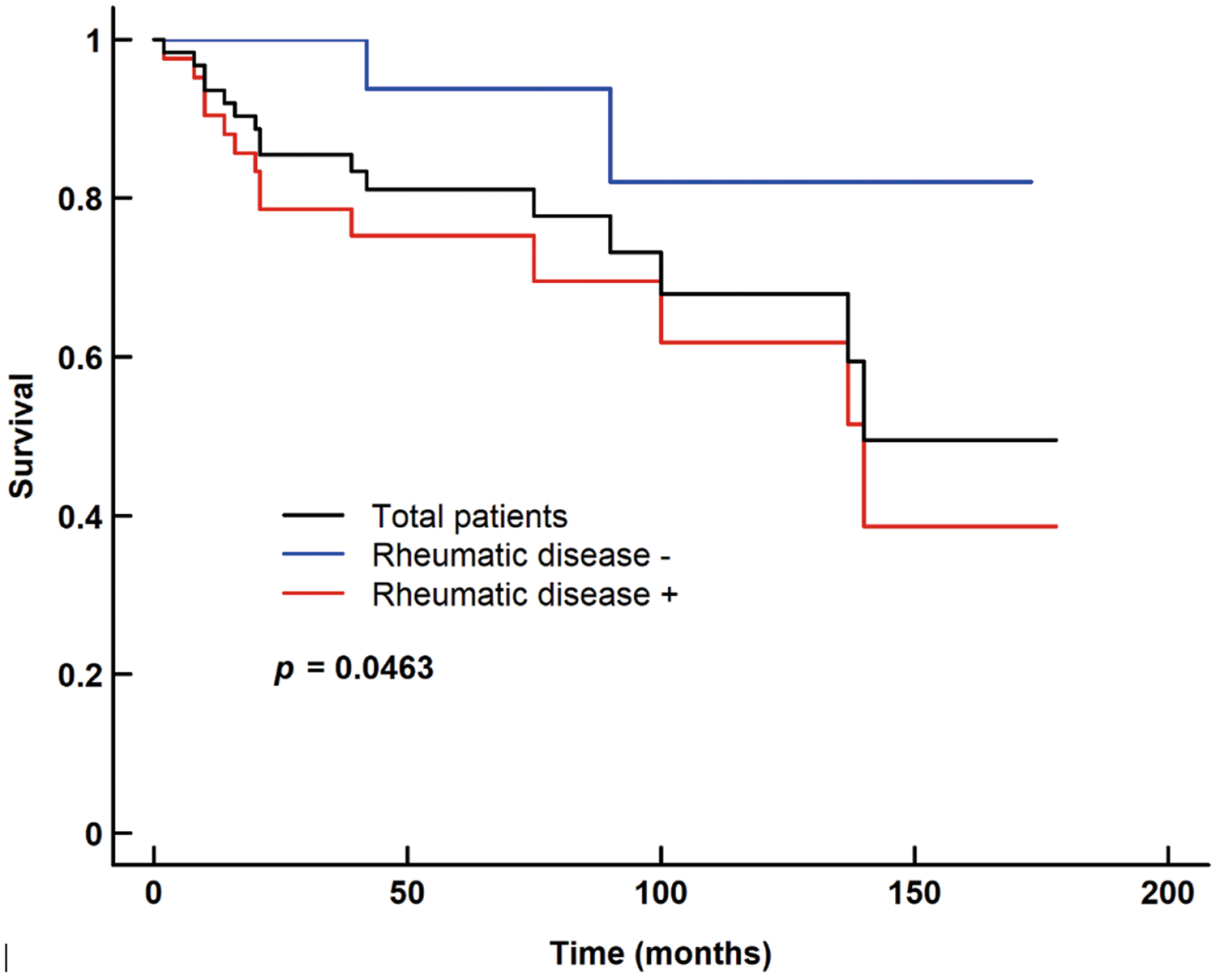
Kaplan–Meier survival curves of symptom-free ischemia.

In the Kaplan–Meier analysis, the 5-year and 10-year symptom-free rates in the total study population were 81.3% and 68.0%, respectively. The 5-year and 10-year symptom-free rates were lower in patients with rheumatic disease than in those without rheumatic disease (75.3% vs. 94.1% at 5 years; 61.8% vs. 82.4% at 10 years), with statistically significant differences between the two groups, as observed after performing the log-rank test (I = 0.046) (Fig. 1) (Supplementary Table S1).

## Discussion

Treatment of chronic hand ischemia is difficult for the practitioner and patient. The general health condition of these patients is usually relatively poor, especially in those with rheumatic disease. Furthermore, a patient’s desire to pursue active treatments may be low because of the psychosocial distress resulting from the chronicity of the disease. Therefore, surgical treatment options for chronic hand ischemia have rarely been reported in the literature. For this reason, many patients do not undergo active management but only observation. To the best our knowledge, the only recent large series on outcomes of bypass surgery using the saphenous vein in below-the-elbow arterial atherosclerotic occlusive disease was reported by Cheun et al.^22^. In their report, open (bypass surgery) and endovascular intervention showed a high success rate and the incidence of major amputation decreased.

However, if neglected, chronic hand ischemia can have detrimental effects on a patient’s daily life, occupation, and quality of life. Patients can experience difficulty with hand motion, severe pain, and may even need to undergo multiple digit amputations. Therefore, practitioners must offer patient-specific treatments, and, if necessary, active surgical management should be considered.

The authors have investigated proper treatment strategies based on patient discomfort levels and angiographic findings^23^. During this process, we have used various graft sources and have finally concluded that the DIEA is the best graft material for several reasons.

First, long-term patency is the most important factor to consider for grafts. In general, the superior long-term durability and clinical outcomes of arterial grafts compared with those of venous grafts is widely accepted, especially for coronary artery bypass grafting^24–27^. The excellent long-term patency of the DIEA has been demonstrated^28,29^. Although, to our knowledge, no study has compared the use of arterial and venous grafts in chronic hand ischemia cases, we preferred the DIEA as the graft based on the findings of the aforementioned studies.

Second, the muscular branches of the DIEA are suitable for various forms of distal anastomoses. Typically, the palmar arch and common digital artery can simultaneously be reconstructed using the Y-shaped graft. As the proximal portion of the DIEA has a similar diameter to the radial and ulnar arteries, the muscular branches also have similar diameters to the common digital artery, facilitating effective microvascular anastomoses.

Third, venous grafts, such as cephalic or saphenous vein grafts, have been widely used in previous studies^20,30–32^. In fact, we generally use cephalic vein grafts for short segments of radial artery or digital artery reconstructions as well. However, venous grafts are highly likely to leave long scars in visible areas. In addition, superficial veins have the potential for unknown injuries. However, in DIEA grafts, long grafts can be harvested with only a 5-cm incision on the abdomen, and, if the patient has no surgical history in the abdomen, the artery can be collected easily without trauma.

Fourth, promising histological characteristics of the DIEA have been revealed in previous studies. The DIEA is thinner than more muscular arteries, such as the radial or gastroepiploic arteries, and has a thicker media compared to the internal mammary artery. These characteristics allow this type of graft to maintain its strength while being less susceptible to ischemia^33^. In addition, in an ex vivo study, the DIEA showed weak responses to vasoconstrictors, such as noradrenaline, phenylephrine, and serotonin, with strong responses to vasodilators, including acetylcholine, substance P, and bradykinin^34^, which can be advantageous for creating a physiologically favorable graft.

It is important to note that there are other sources of arterial grafts, including the thoracodorsal artery and the lateral circumflex femoral artery (LCFA). However, the thoracodorsal artery and its branches have relatively larger diameters compared to the palmar arch and the common digital artery. Furthermore, to harvest the LCFA, a long scar is unavoidable. Moreover, the LCFA is susceptible to atherosclerosis and degenerative changes. In contrast, the DIEA is relatively spared from these pathologic changes, suggesting that it is a better arterial graft source. In addition, the DIEA has relatively few anatomical variations. Only the amount of intramuscular portion of the artery varied, which could be solved by intramuscular dissection. Therefore, our first choice was the DIEA, and only if it was impossible, alternative graft sources, such as the thoracodorsal artery or lateral circumflex femora artery, were considered.

Our study also showed that rheumatic disease is a key factor in chronic hand ischemia cases. Both occurrence and recurrence were closely related to rheumatic disease, as has been reported in a previous study^35^. In the present study, long-term symptom-free rates after DIEA grafts were significantly higher in cases without rheumatic disease, with a 10-year symptom-free rate of 68% in patients with rheumatic disease. We do not think that the patency or the effects of DIEA grafts can be permanently sustained in patients with rheumatic disease, as these grafts are affected by the progression of the systemic disease. However, during the period, in which the effect of the surgery is maintained, the patient can return to work and maintain a better quality of life, which are important factors to consider.

**Figure 2:**
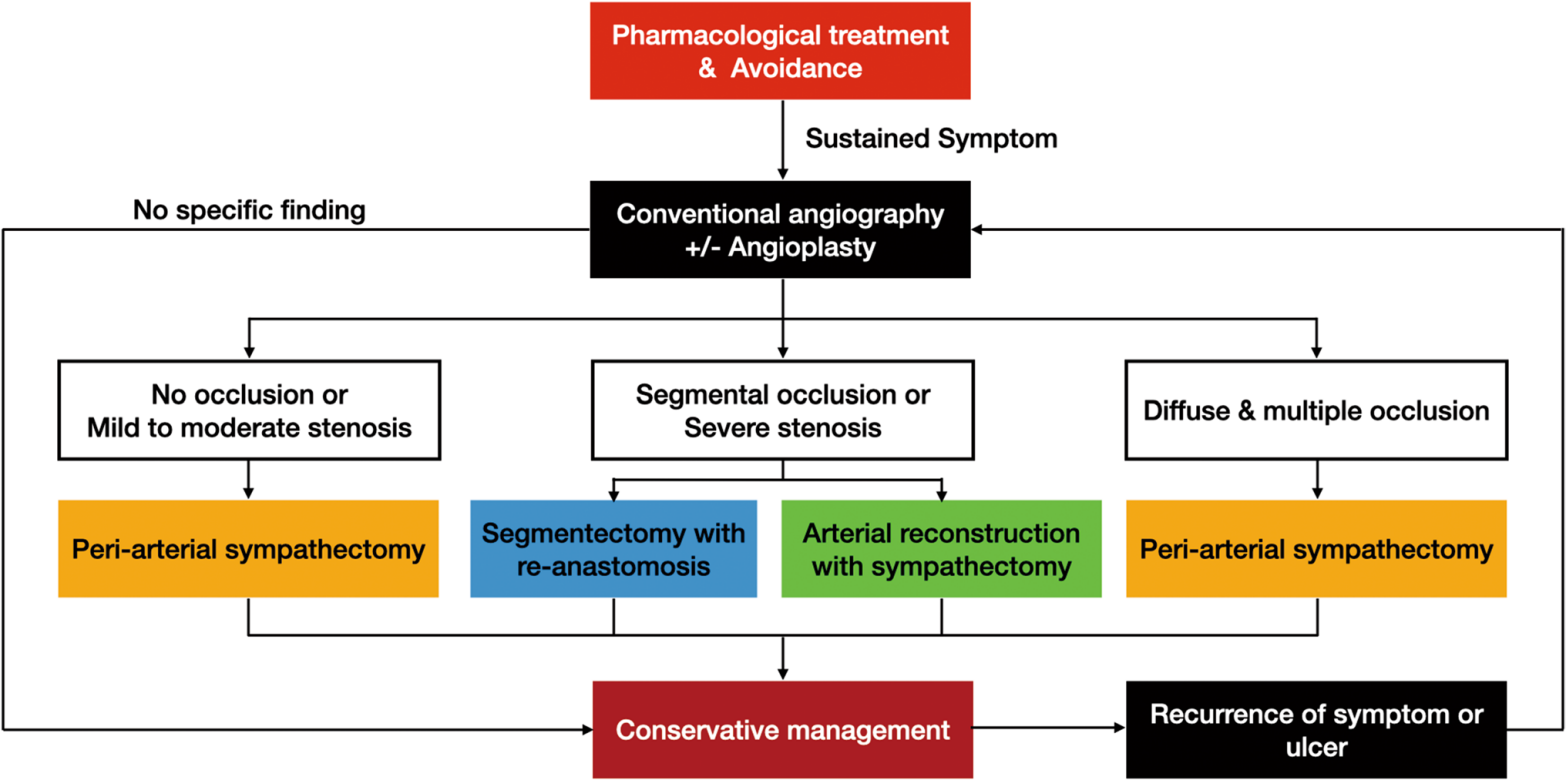
Algorithmic approach to surgical management of chronic hand ischemia.

The progression rate and severity of hand ischemia also affected the DIEA graft results. The duration from symptom onset to the operation was significantly longer in the recurred group. A previous sympathectomy, dual radial and ulnar artery grafts, and a concomitant amputation or debridement were significant risk factors for recurrence in the univariate Cox regression analysis. A higher sympathectomy level (including the CPDA and PPDA) and a longer graft length were identified as risk factors for recurrence in the multivariate Cox regression analysis. Although results from the various analyses were not consistent, more severe preoperative conditions had a tendency to be associated with worse results. Therefore, we recommend that an interpositional arterial graft should be performed before an occlusion has progressed to the CPDA level. Timely management is important for avoiding ischemic consequences, including fingertip necrosis and amputation. Therefore, we applied an algorithmic approach for obtaining a more precise diagnosis and more timely management (Fig. 2)^15^.

The most common procedure for chronic hand ischemia cases is bypass vein grafting^36,37^. There are several differences between arterial interpositional graft and bypass vein graft other than the graft source. For bypass graft, microvascular anastomosis is mostly performed in end-to-side manner^15^. It is technically more difficult compared to end-to-side anastomosis that is performed for the interpositional graft. In addition, vascular flow can be more unreliable or weak than straightforward flow after end-to-end anastomosis because the inflow vessel is not normal in most cases. However, the objective comparison between the two methods was impossible because several aspects, including the underlying diseases and the bypass level, were different compared to those of the patients assessed in previous studies. Moreover, long-term result or finding for recurrence was unavailable. In a recent systematic review, all patients that underwent bypass surgery showed pain improvement and 92.7% of patients showed ulcer healing, which was similar with our result. However, there were no data related to long-term follow-up to assess recurrence trends^15^.

**Figure 3:**
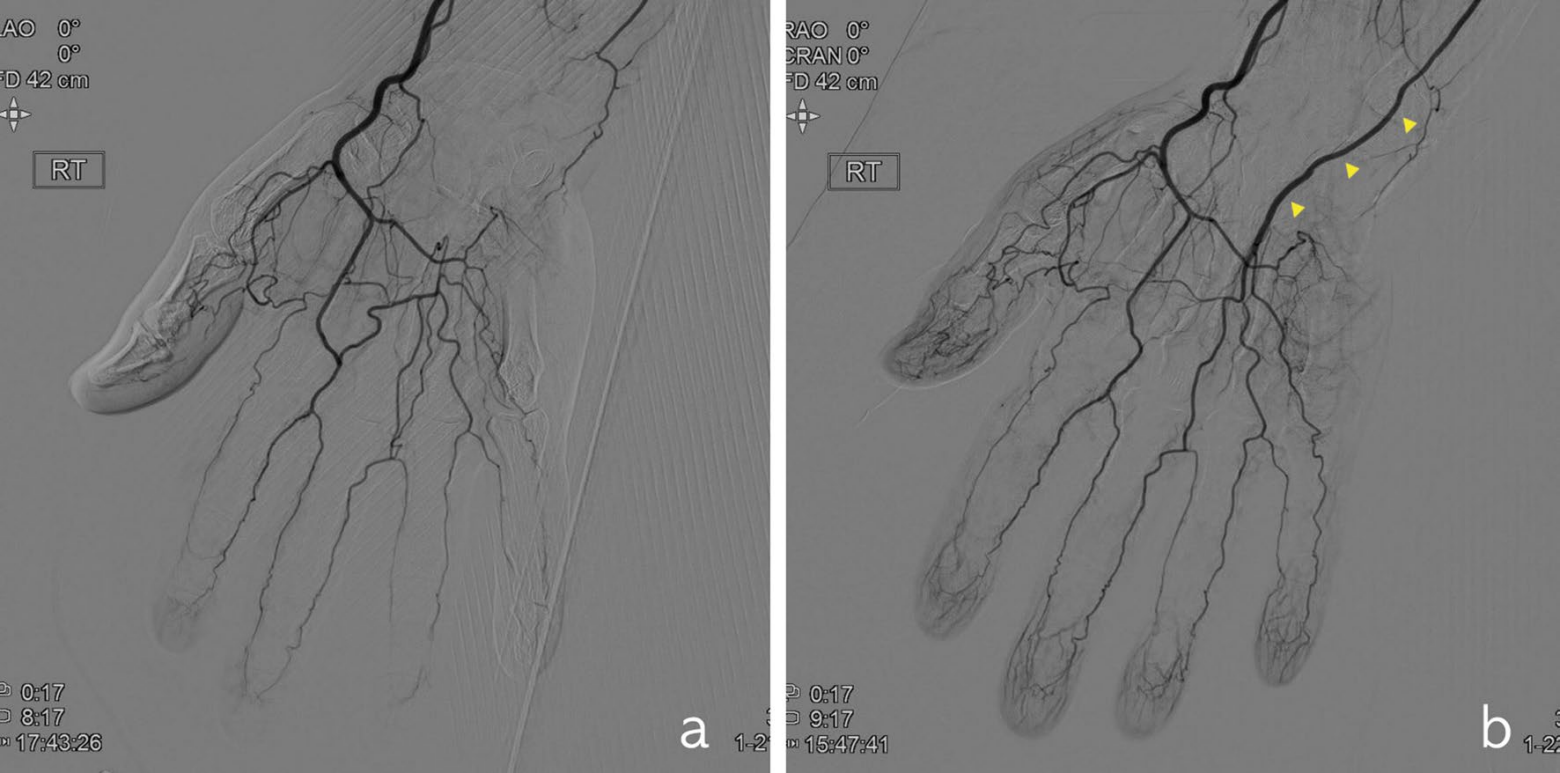
Conventional angiography findings. (**a**) Occlusion of the ulnar artery and decreased blood flow in the superficial palmar arch were observed. A Y-shaped DIEA interpositional graft was anastomozed to three locations, including the proximal ulnar artery, the superficial palmar arch, and the 3^rd^ common palmar digital artery; (**b**) a 12-month postoperative angiogram shows patent blood flow in the graft (yellow arrows) and increased blood flow in the digital arteries.

This study had several limitations. First, given its retrospective design, bias may exist because of inconsistencies in the study population. In this study, no control group that used a vein graft was employed. Our focus is not the superiority of DIEA grafts to vein grafts but the long-term effect of the DIEA graft itself. In addition, the severity, type, and effects of rheumatic disease were not evaluated in detail. Moreover, the criteria for recurrence were not precise and were based on a patient's symptoms rather than on the direct evaluation of the patency of the vessel graft itself. As conventional angiography is an invasive procedure, we did not perform it for routine follow-up examinations but only in select cases who required additional workups (Fig. 3b). In addition, even in patients in whom vessel grafts were occluded because of progression of the underlying disease, the effect of surgery was considered to be maintained if symptomatic improvement was sustained. In our experience, patients with concomitant digital arterial occlusions tend to be more susceptible to symptomatic recurrence. However, this trend was not evaluated objectively. Finally, the number of included cases was relatively small, which limited our ability to perform statistical analyses. This small sample size was related to the rarity of patients with chronic hand ischemia. Furthermore, long-term follow-up examination is not easy in these patients because of aggravation of the systemic disease. However, to the best of our knowledge, there have been no previous long-term follow-up and large-series studies related to interpositional grafting or peripheral arterial bypass grafting in patients with chronic hand ischemia. Further follow-up examination periods and analyses based on this study may provide more objective results related to the management of chronic hand ischemia.

## Methods

### Study population

This retrospective study was approved by Institutional Review Board (IRB) of Hanyang University Hospital (IRB No.: 2020–09-014). All methods were carried out in accordance with relevant guidelines and ethical regulations. Informed consent from participant was waived by IRB of Hanyang University Hospital (IRB No.: 2020-09-014) because this study was a non-interventional retrospective design and analyzed anonymously. Patients who had undergone surgery involving an interpositional DIEA graft combined with periarterial sympathectomy for chronic hand ischemia from March 2003 to February 2019 were included. Patients with insufficient medical records or those who underwent follow-up periods < 12 months were excluded. In total, 62 cases had data collected from three overall categories, including demographic, operation-related, and result-related variables. In patients who had undergone DIEA grafting in bilateral hands, each hand was analyzed separately.

### Preoperative assessment

A hand-held Doppler examination was routinely performed. Surgical treatment was considered for patients whose chronic hand ischemia did not respond to medication for at least 3 months or for patients with severe ischemic symptoms, including pain, cold intolerance, or ulceration. Calcium channel blockers (nifedipine), aspirin, and prostacyclin analogs (beraprost sodium) were used as vasodilatory and anti-thrombotic pharmacological agents in these patients^4^.

Conventional angiography was performed to estimate the whole blood flow and to identify stenotic lesions. If complete or near-complete occlusion not resolvable by sympathectomy was noted, an interpositional arterial graft was considered (Fig. 3a).

### Surgical technique

Based on angiographic findings, an inverted, J-shaped incision from the wrist level to the palmar crease was made along the course of the ulnar or radial artery to the palmar arch. A periarterial sympathectomy was performed by stripping the adventitia of the blood vessel through the sympathetic nerve innervating around the blood vessel under a surgical microscope. The range of the periarterial sympathectomy depended on the patient’s condition, which was determined by angiographic and intraoperative findings. In patients with severe disease, sympathectomy was performed for the CPDA and PPDA. If a sympathectomy of the PPDA was required, an additional incision was made. If pathologic fibrotic bands or protruding structures were encountered during the dissection, decompression was performed by adhesiolysis and removal of structures. These findings were frequently discovered in Guyon’s canal of the carpal bones.

After identifying the extent of the segmental stenosis or occlusion of the artery, we performed segmentectomy of the occluded segment and checked for blood patency at the proximal and distal ends. If the length of the excised segment was short, a direct reanastomosis was performed. However, in most cases, an interpositional graft using the DIEA was needed, and the DIEA graft was harvested according to the length and configuration of the defect. The DIEA graft harvest was performed using a transverse incision approximately 5 cm in length on the lower abdomen on the opposite side from the hand with a two-team approach. In some ulnar artery cases, the defect reached to the superficial palmar branch and the CPDA. In these patients, the muscular branch of the DIEA was further dissected, and the graft was harvested with a Y-shape. End-to-end anastomoses were performed and, after pulsation of the graft was confirmed, wound closure was performed (Supplementary Fig. S2, Supplementary Fig. S3, Supplementary Video 1).

### Postoperative management and outcome measurement

A dorsal splint with 20 degrees of wrist flexion was applied to immobilize the wrist and to protect the anastomotic site for 1 week. To increase vascularity and vasodilation, 10 mcg of prostaglandin E1 was administered intravenously for 1 week postoperatively. In addition, for patients with thrombotic tendencies, heparin was administered intravenously at a dosage of 800 IU/h during the first 6 h and at 400 IU/h for 7 days postoperatively. After the administration of intravenous agents was completed, calcium channel blocker, aspirin, and prostacyclin analogs were used as oral medications.

Recurrence was confirmed based on patient symptoms, including intractable pain or ulceration. If definite symptomatic recurrence occurred at the same digit or the same location, it was defined as a recurrence. Symptoms were evaluated meticulously to distinguish graft occlusion from aggravation of systemic disease or Raynaud’s phenomenon. In addition, a hand-held Doppler examination was performed as an adjunctive evaluation tool. If ischemic symptoms recurred and abnormal findings on the Doppler examination were detected, evaluation via conventional angiography was considered.

### Statistical analysis

To identify variables influencing recurrence, the patients were divided into the recurred and non-recurred groups during the follow-up period. Pearson’s chi-squared test, Fisher's exact test, and the Wilcoxon rank-sum test were used to analyze demographic, operation-related, and result-related variables of patients. Univariate and multivariate Cox regression analyses were performed to identify risk factors for the recurrence of ischemic symptoms, with results expressed as HRs with 95% CIs. A backward selection model was applied for the multivariate analysis. Kaplan–Meier analysis was performed to compare the rates of recurrence based on the presence of rheumatic disease. Statistical analyses were performed using SAS version 9.4 (SAS Institute Inc., Cary, North Carolina, USA). A *p*-value < 0.05 was considered statistically significant.

## Supplementary Information

Supplementary Video 1.

Supplementary Information 1.

Supplementary Information 2.

Supplementary Information 3.

Supplementary Information 4.

Supplementary Information 5.

## Data Availability

The data analyzed in this study is available from the corresponding author on reasonable request.
