# Supplementary figures and images for: Long-term results of ulnar and radial reconstruction with interpositional grafting using the deep inferior epigastric artery for chronic hand ischemia
Source: Sci Rep. 2021 Nov 30;11:23185. doi: 10.1038/s41598-021-02530-6 (PMC8633384; doi:10.1038/s41598-021-02530-6)

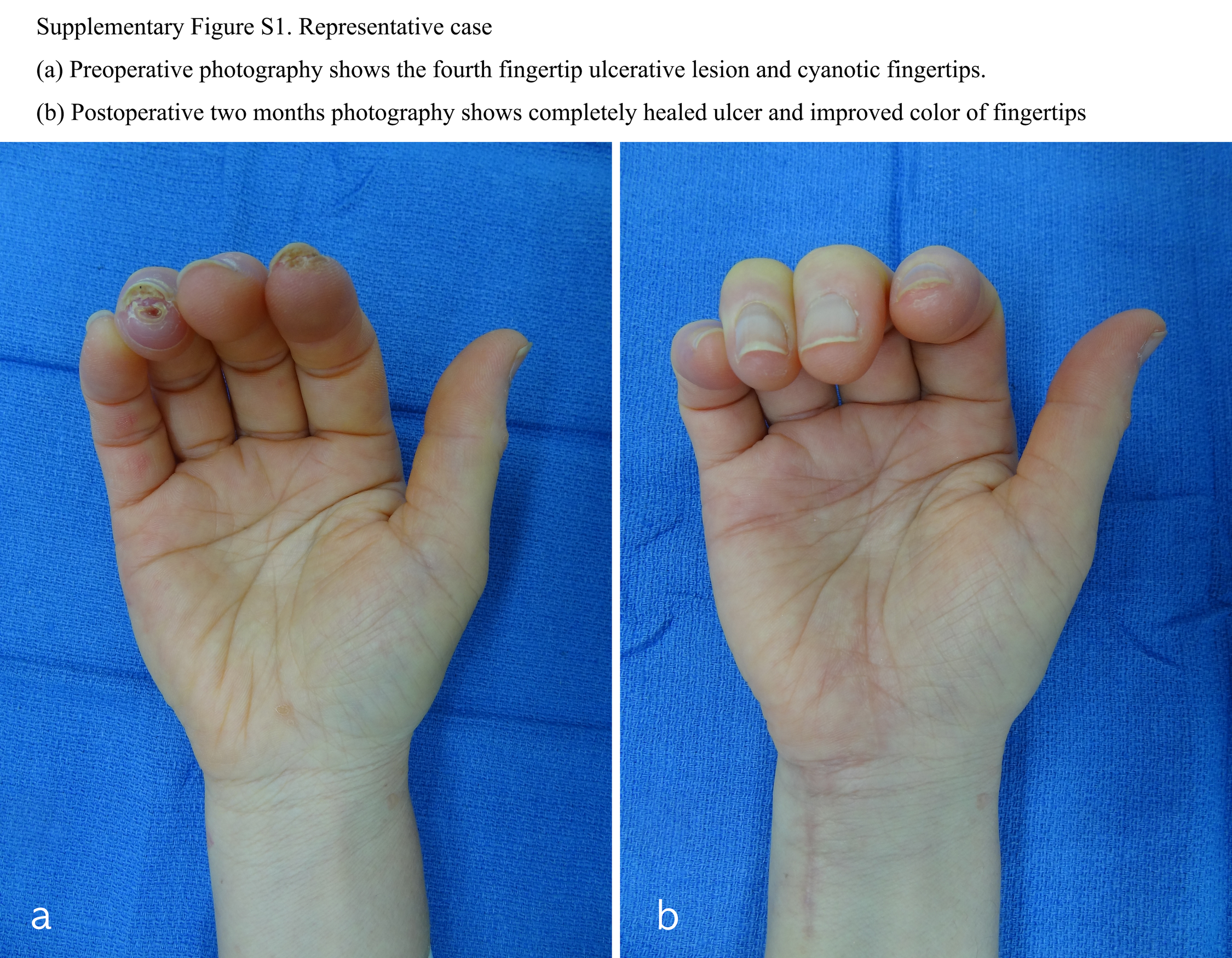

Supplement: Supplementary file 2 — Supplementary Information 1. [file 41598_2021_2530_MOESM2_ESM.tif]

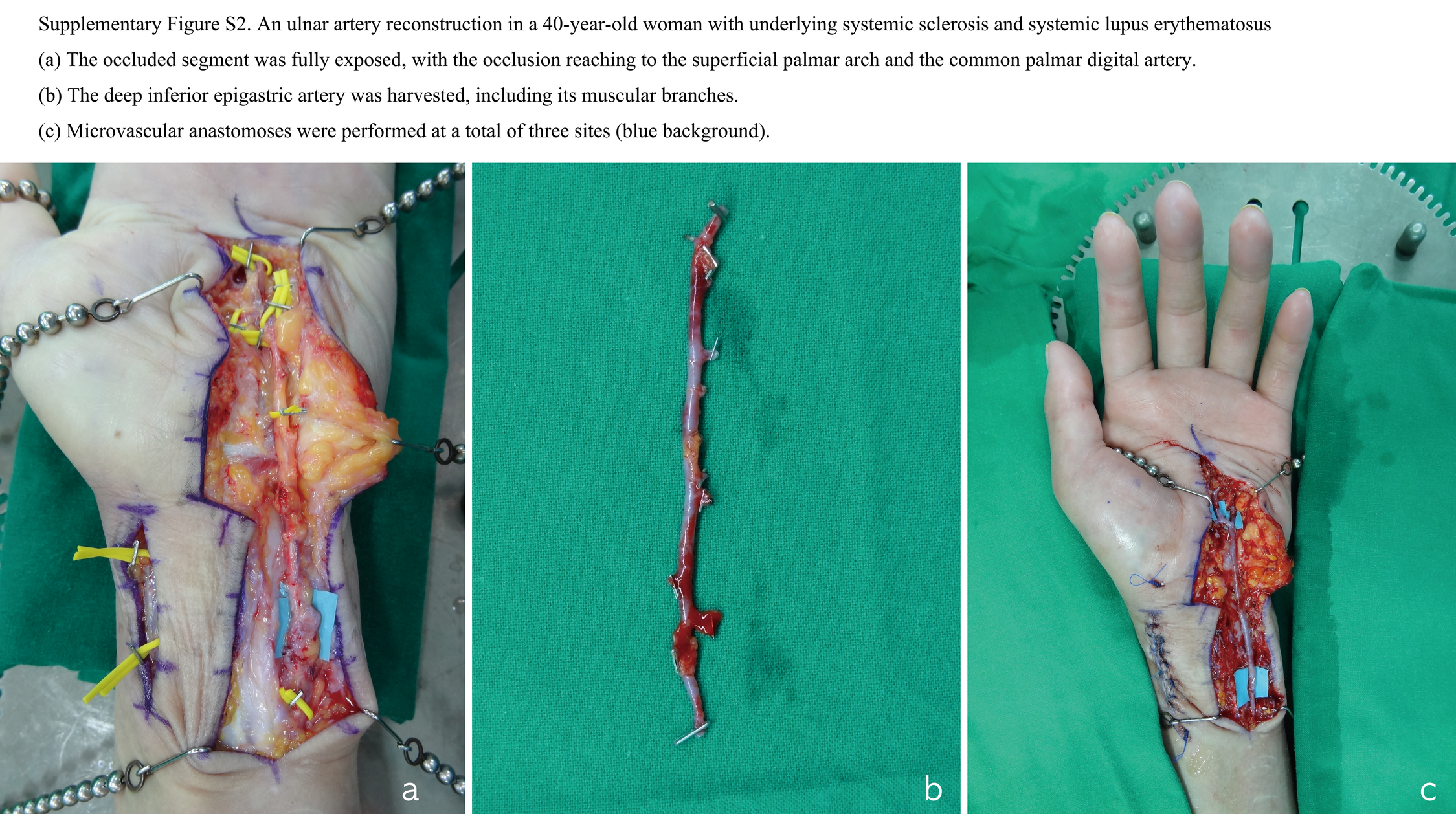

Supplement: Supplementary file 3 — Supplementary Information 2. [file 41598_2021_2530_MOESM3_ESM.tif]

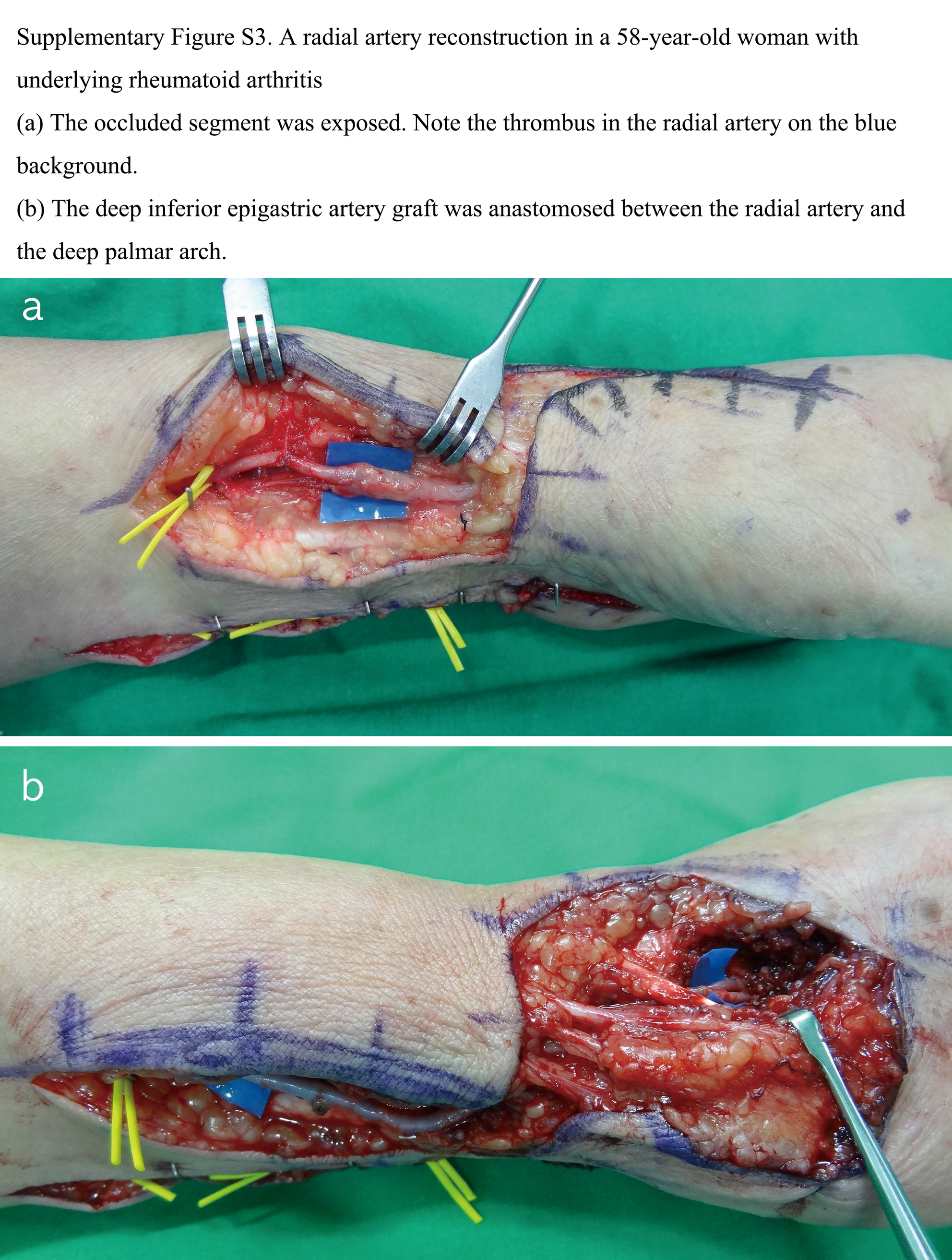

Supplement: Supplementary file 4 — Supplementary Information 3. [file 41598_2021_2530_MOESM4_ESM.tif]
