# Supplementary material for: Long-term results of ulnar and radial reconstruction with interpositional grafting using the deep inferior epigastric artery for chronic hand ischemia
Source: Sci Rep. 2021 Nov 30;11:23185. doi: 10.1038/s41598-021-02530-6 (PMC8633384; doi:10.1038/s41598-021-02530-6)
Supplement: Supplementary file 5 — Supplementary Information 4. [file 41598_2021_2530_MOESM5_ESM.docx]

**Supplementary Material**

Supplementary Figure S1. Representative case

(a) Preoperative photography shows the fourth fingertip ulcerative lesion and cyanotic fingertips. (b) Postoperative two months photography shows completely healed ulcer and improved color of fingertips

Supplementary Figure S2. An ulnar artery reconstruction in a 40-year-old woman with underlying systemic sclerosis and systemic lupus erythematosus

(a) The occluded segment was fully exposed, with the occlusion reaching to the superficial palmar arch and the common palmar digital artery. (b) The deep inferior epigastric artery was harvested, including its muscular branches. (c) Microvascular anastomoses were performed at a total of three sites (blue background).

Supplementary Figure S3. A radial artery reconstruction in a 58-year-old woman with underlying rheumatoid arthritis

(a) The occluded segment was exposed. Note the thrombus in the radial artery on the blue background. (b) The deep inferior epigastric artery graft was anastomosed between the radial artery and the deep palmar arch.

Supplementary Video 1. Good visible pulsations after microvascular anastomoses. A Y-shaped DIEA interpositional graft was anastomosed to three locations, including the proximal ulnar artery, the superficial palmar arch, and the 3^rd^ common palmar digital artery.

Supplementary Table S1. Life-table of the Kaplan-Meier analysis
