# Supplementary material for: Long-term results of ulnar and radial reconstruction with interpositional grafting using the deep inferior epigastric artery for chronic hand ischemia
Source: Sci Rep. 2021 Nov 30;11:23185. doi: 10.1038/s41598-021-02530-6 (PMC8633384; doi:10.1038/s41598-021-02530-6)
Supplement: Supplementary file 6 — Supplementary Information 5. [file 41598_2021_2530_MOESM6_ESM.docx]

Supplementary Table S1. Life-table of the Kaplan-Meier analysis

| Interval | Total (n = 62) | | | | | Rheumatic disease = Yes  (n = 42) | | | | | Rheumatic disease = No  (n = 20) | | | | |
| --- | --- | --- | --- | --- | --- | --- | --- | --- | --- | --- | --- | --- | --- | --- | --- |
|  | Number  Failed | Number Censored | Survival | Failure | Survival Standard Error | Number  Failed | Number Censored | Survival | Failure | Survival Standard Error | Number  Failed | Number Censored | Survival | Failure | Survival Standard Error |
| [0, 12) | 4 | 0 | 1.000 | 0.000 | 0.000 | 4 | 0 | 1.000 | 0.000 | 0.000 | 0 | 0 | 1.000 | 0.000 | 0.000 |
| [12, 24) | 5 | 0 | 0.936 | 0.065 | 0.031 | 5 | 0 | 0.905 | 0.095 | 0.045 | 0 | 0 | 1.000 | 0.000 | 0.000 |
| [24, 36) | 0 | 8 | 0.855 | 0.145 | 0.045 | 0 | 6 | 0.786 | 0.214 | 0.063 | 0 | 2 | 1.000 | 0.000 | 0.000 |
| [36, 48) | 2 | 8 | 0.855 | 0.145 | 0.045 | 1 | 6 | 0.786 | 0.214 | 0.063 | 1 | 2 | 1.000 | 0.000 | 0.000 |
| [48, 60) | 0 | 5 | 0.813 | 0.187 | 0.051 | 0 | 3 | 0.753 | 0.247 | 0.069 | 0 | 2 | 0.941 | 0.059 | 0.057 |
| [60, 72) | 0 | 3 | 0.813 | 0.187 | 0.051 | 0 | 1 | 0.753 | 0.247 | 0.069 | 0 | 2 | 0.941 | 0.059 | 0.057 |
| [72, 84) | 1 | 8 | 0.813 | 0.187 | 0.051 | 1 | 6 | 0.753 | 0.247 | 0.069 | 0 | 2 | 0.941 | 0.059 | 0.057 |
| [84, 96) | 1 | 2 | 0.778 | 0.222 | 0.060 | 0 | 0 | 0.695 | 0.305 | 0.084 | 1 | 2 | 0.941 | 0.059 | 0.057 |
| [96, 108) | 1 | 2 | 0.732 | 0.268 | 0.072 | 1 | 0 | 0.695 | 0.305 | 0.084 | 0 | 2 | 0.824 | 0.177 | 0.121 |
| [108, 120) | 0 | 0 | 0.680 | 0.320 | 0.084 | 0 | 0 | 0.618 | 0.382 | 0.105 | 0 | 0 | 0.824 | 0.177 | 0.121 |
| [120, 132) | 0 | 4 | 0.680 | 0.320 | 0.084 | 0 | 2 | 0.618 | 0.382 | 0.105 | 0 | 2 | 0.824 | 0.177 | 0.121 |
| [132, 144) | 2 | 1 | 0.680 | 0.320 | 0.084 | 2 | 1 | 0.618 | 0.382 | 0.105 | 0 | 0 | 0.824 | 0.177 | 0.121 |
| [144, 156) | 0 | 0 | 0.499 | 0.502 | 0.126 | 0 | 0 | 0.393 | 0.607 | 0.143 | 0 | 0 | 0.824 | 0.177 | 0.121 |
| [156, 168) | 0 | 2 | 0.499 | 0.502 | 0.126 | 0 | 1 | 0.393 | 0.607 | 0.143 | 0 | 1 | 0.824 | 0.177 | 0.121 |
| [168, 180) | 0 | 3 | 0.499 | 0.502 | 0.126 | 0 | 2 | 0.393 | 0.607 | 0.143 | 0 | 1 | 0.824 | 0.177 | 0.121 |
